# Xylose: absorption, fermentation, and post-absorptive metabolism in the pig

**DOI:** 10.1186/s40104-017-0226-9

**Published:** 2018-01-07

**Authors:** Nichole F. Huntley, John F. Patience

**Affiliations:** 1 Department of Animal Science, 213 Kildee Hall, Iowa State University, Ames, 50011 IA USA; 2 Department of Animal Science, 201B Kildee Hall, Iowa State University, Ames, 50011 IA USA

**Keywords:** Pentose utilization, Swine, Threitol, Xylose absorption, Xylose metabolism

## Abstract

Xylose, as β-1,4-linked xylan, makes up much of the hemicellulose in cell walls of cereal carbohydrates fed to pigs. As inclusion of fibrous ingredients in swine diets continues to increase, supplementation of carbohydrases, such as xylanase, is of interest. However, much progress is warranted to achieve consistent enzyme efficacy, including an improved understanding of the utilization and energetic contribution of xylanase hydrolysis product (i.e. xylooligosaccharides or monomeric xylose). This review examines reports on xylose absorption and metabolism in the pig and identifies gaps in this knowledge that are essential to understanding the value of carbohydrase hydrolysis products in the nutrition of the pig. Xylose research in pigs was first reported in 1954, with only sporadic contributions since. Therefore, this review also discusses relevant xylose research in other monogastric species, including humans. In both pigs and poultry, increasing purified D-xylose inclusion generally results in linear decreases in performance, efficiency, and diet digestibility. However, supplementation levels studied thus far have ranged from 5% to 40%, while theoretical xylose release due to xylanase supplementation would be less than 4%. More than 95% of ingested D-xylose disappears before the terminal ileum but mechanisms of absorption have yet to be fully elucidated. Some data support the hypothesis that mechanisms exist to handle low xylose concentrations but become overwhelmed as luminal concentrations increase. Very little is known about xylose metabolic utilization in vertebrates but it is well recognized that a large proportion of dietary xylose appears in the urine and significantly decreases the metabolizable energy available from the diet. Nevertheless, evidence of labeled D-xylose-1-^14^C appearing as expired ^14^CO_2_ in both humans and guinea pigs suggests that there is potential, although small, for xylose oxidation. It is yet to be determined if pigs develop increased xylose metabolic capacity with increased adaptation time to diets supplemented with xylose or xylanase. Overall, xylose appears to be poorly utilized by the pig, but it is important to consider that only one study has been reported which supplemented D-xylose dietary concentrations lower than 5%. Thus, more comprehensive studies testing xylose metabolic effects at dietary concentrations more relevant to swine nutrition are warranted.

## Background

**Fig. 1:**
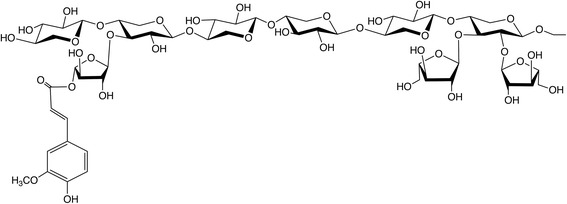
β-1,4-linked D-arabinoxylan. *α*-1,3 L-arabinose residues are linked on position ^3^O, or ^2^O and ^3^O if di-substituted, of D-xylose unit. Ferulic acid is linked on position ^5^O from arabinose

Xylose, as a major constituent of plant xylan polymers, is one of the most abundant carbohydrates on the earth, second only to glucose [1, 2]. This abundant pentose sugar, along with arabinose, makes up a majority of the hemicellulose backbone as arabinoxylan in the cell walls of cereal grains fed to pigs [3]. Cereal arabinoxylans (also known as pentosans) are composed of a linear β-1,4-linked xylose backbone which may be substituted at the 2’-OH and/or 3’-OH, generally with single arabinose residues (Fig. 1) [1]. In contrast to α-linked starch, which is hydrolyzed to glucose by endogenous enzymes, β-linkages in arabinoxylan and other non-starch polysaccharides (NSP) must be degraded by microbial enzymes. Thus, pigs cannot derive energy directly from arabinoxylans. The antinutritional effects of arabinoxylans and other NSP have been previously reviewed [4–6].

Generally, increasing dietary fiber concentration reduces diet energy density and dry matter (DM) digestibility resulting in impaired feed efficiency and growth performance. Exogenous carbohydrases, such as xylanase, have been developed and utilized to combat these effects. Assessment of enzyme efficacy is commonly based on animal growth, nutrient utilization, feed efficiency, and carcass quality; responses to carbohydrase supplementation are often quite variable [7, 8]. The large proportion of arabinoxylan in the hemicellulose fraction of cereal grains and coproducts makes xylanase supplementation of great interest in swine nutrition [9, 10]. While greater progress is needed to achieve consistent enzyme efficacy, it is yet to be determined how the products of xylanase hydrolysis will be utilized by the pig and to what extent these products may contribute to overall energy balance.

**Fig. 2:**
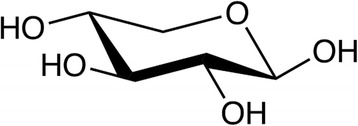
D-xylose

Xylanase hydrolysis products have not been well-studied in vivo, but an understanding of enzyme function indicates that a mix of oligosaccharides, disaccharides, and monomeric pentose sugars, such as xylose (Fig. 2), may be released through the combined actions of xylanases with various activities [11–13]. Complete arabinoxylan degradation to monosaccharides requires multiple hemicellulases [14]. Typical growing pig diets will contain 3 - 5% polymeric xylose which, if released as free xylose, could significantly impact the nutrition of growing pigs. Yet, the current understanding of xylose metabolism and potential contribution to dietary energy is vague at best. This review examines published, peer-reviewed research on xylose absorption and metabolism in the pig and identifies gaps in this knowledge that are essential to understanding the value of carbohydrase hydrolysis products for swine nutrition. Xylose research in pigs began with initial observations by Wise et al. [15] in 1954 and has been far and few between ever since. Therefore, this review will also discuss relevant xylose absorption, metabolism, and fermentation research in other monogastric species, including humans. Furthermore, xylose effects on glucose and energy balance in monogastric animals are considered.

## Effects of xylose on animal performance

In both pigs and poultry, increasing the dietary inclusion of D-xylose generally results in a linear decrease in average daily gain (ADG), feed intake, feed efficiency, and excreta DM content [15–19]. Linear decreases in the apparent total tract digestibility of DM, energy, and nitrogen were reported in broilers consuming dietary D-xylose concentrations of 0%, 5%, and 15% [19]. Exceptions exist in poultry with Longstaff et al. [20] who reported no significant differences at 5% or 20% dietary D-xylose inclusion and in pigs where ADG was not reduced when a basal diet was supplemented with 10% D-xylose compared to 5% D-glucose [21]. However, xylose generally imparts negative performance responses that, in some cases, have been severe. Two-week-old pigs fed purified diets containing 18.7% or 37.4% D-xylose had drastically decreased feed intake and ADG compared to pigs fed 37.4% D-glucose [15]. The authors also noted decreased physical activity in xylose-fed pigs along with vomiting, diarrhea, cataract formation, and nephritis in pigs fed the highest xylose diet [15].

Importantly, these drastic xylose effects on animal performance are only observed when purified D-xylose is supplemented, not with xylanase supplementation [22–24]. This distinction could be explained if exogenous xylanase releases only small quantities of monomeric xylose in vivo, as compared to the levels fed in the above-mentioned studies. Based on the NSP composition of fibrous ingredients fed to growing pigs, most diets rarely contain more than 5% xylose [3, 9, 10, 25], regardless of the degree of polymerization. Furthermore, only a small proportion of the polymeric xylose would likely be hydrolyzed to its monomeric components [26]. Even if 100% of the xylose molecules are liberated through exogenous enzyme supplementation, this level is unlikely to compromise pig health. However, the minimum level of xylose required to impact pig health has not yet been defined empirically.

## Xylose absorption and disappearance

In pigs, xylose has a high disappearance rate in the small intestine, ranging from 96 to 99% when supplemented as purified D-xylose [17, 21, 27, 28]. Similarly, xylose disappearance in poultry is very high (93.5 – 97.8%) when supplemented at 5% of diet DM; the rate of disappearance from the gut declined as the quantity of xylose in the diet increased: 91.9% at 20% and 87.3% at 40% dietary D-xylose [17, 20]. Although it is possible that a portion of the xylose disappearance is due to microbial fermentation in the small intestine, there is empirical evidence that xylose is also readily absorbed in the duodenum and proximal jejunum in rats [29, 30]. This is assumed to be similar in pigs and poultry based on disappearance prior to the cecum [27, 28, 31, 32]. However, xylose mucosal transport throughout the entire digestive tract, including the colon, has been described [31]. Providing purified D-xylose in the diet at high levels (> 20%), also decreases ileal digestibility of DM, organic matter, gross energy and nitrogen in pigs [21, 27].

Mechanisms of xylose absorption have yet to be fully characterized in pigs and poultry. Passive diffusion was initially reported [33], but most research indicates the existence of a sodium-dependent active transport system similar to that described for glucose and amino acids [27, 31, 32, 34]. D-xylose in rat diets induced expression of sodium-linked glucose transporter mRNA [35] suggesting at least some xylose is carried by this transporter and that competitive binding with glucose may occur. [34]. Xylose did not affect mRNA abundance of either *GLUT2* or *GLUT5* [35]. An active transport mechanism is further supported by data from equine and rabbit jejunal tissue which accumulated xylose against a concentration gradient when incubated in a 1-mmol/L D-xylose solution [36]. However, when the tissues were incubated in a 5-mmol/L D-xylose solution, no accumulation was reported.

Freeman’s data in horses and rabbits [36] suggest that an active transport system may exist for xylose although it has a low affinity and is easily saturated. If this system does exist in swine, the proportion of xylose which is actively transported is probably very low. This hypothesis may relate to the considerably slower rates of xylose absorption compared to glucose [30]. Furthermore, because the transportation system becomes saturated at low xylose concentrations, absorption would be expected to continue to occur through diffusion given the high xylose disappearance rate in the small intestine [36]. In humans, however, xylose is absorbed by sodium-independent passive diffusion [37, 38] and no inhibitory effects of glucose have been observed [38].

Although precise mechanisms of xylose absorption and retention have not been reported for pigs and poultry, based on data in other species, it is clear that xylose is readily absorbed but at a slower rate than galactose and glucose and faster than arabinose [16, 30, 39]. Like most other water-soluble monosaccharides, xylose is then probably transported from the serosal side of the enterocyte to systemic circulation via the portal vein to the liver [40]. However, transporters potentially involved in this transfer have not been described. Once xylose is absorbed into the enterocyte it must be retained. Unlike glucose, reports on xylose phosphorylation are variable and the retention mechanism is unclear. Mucosal homogenates from rats did not demonstrate the ability to phosphorylate xylose [41]. In contrast, 13 and 36% of the xylose accumulated in jejunal tissues during D-xylose incubation was phosphorylated in the equine and rabbit tissues, respectively [36]; xylose phosphorylation following active absorption has also been reported [42].

While absorption mechanisms of xylose may not be fully revealed, its ability to decrease glucose absorption has been more clearly demonstrated. Currently, xylose is of interest in human nutrition as a supplement to suppress postprandial glucose and insulin surges. Xylose has been reported to selectively inhibit sucrase activity in a non-competitive manner [43]. When consumed with a glucose solution or high carbohydrate meal, xylose decreased serum glucose levels up to 30 min post-consumption in one study in humans [44] and up to 120 min in another [45]. Additionally, xylose supplementation decreased insulin area under the curve for up to 90 min compared to a pure glucose solution or high carbohydrate meal [44, 45].

Suppression of the glycemic effect may occur in part through sucrase inhibition as well as through stimulation of glucagon like peptide-1 (GLP-1) secretion. In humans with type 2 diabetes consumption of a 50-g D-xylose solution 40 min prior to a high carbohydrate meal attenuated the postprandial glycemic and insulin responses associated with GLP-1 secretion before the meal [46]. The authors initially postulated that GLP-1 secretion was stimulated in response to short chain fatty acids (SCFA) produced through bacterial fermentation of xylose, confirmed by an increase in hydrogen production in breath samples [46]. However, GLP-1 concentrations began to increase within 20 min of xylose ingestion, suggesting that more direct metabolism of the pentose was also involved [46].

Some researchers have speculated that xylose supplementation would not exert similar effects in a high-fat meal due to the typically lower glycemic response [45], yet positive responses have been demonstrated. In obese mice fed high-fat diets, 5% and 10% D-xylose supplementation attenuated fasting blood glucose concentration back to healthy control levels, compared to mice fed a high-fat diet without xylose [47]. However, in poultry, no effect of xylose supplementation on serum glucose concentration was reported and the responses to increasing dietary xylose concentration on serum insulin were highly variable [19]. This discrepancy may be related to the tight regulation of circulating glucose concentrations in birds [48]. The effects of xylose on glucose absorption and regulation must be considered when determining how xylanase hydrolyzed monomers impact overall energy balance in pigs.

## Xylose fermentation

Results of most experiments with monomeric xylose report almost complete disappearance prior to the terminal ileum; furthermore, increasing dietary xylose inclusion has been reported to increase ileal SCFA flow [27], cecal SCFA concentration [49], and cecal weight [16], indicating some degree of microbial fermentation of xylose in the small intestine. In humans, consumption of a 50-g D-xylose solution was reported to increase breath hydrogen production, a measure of bacterial fermentation, within 40 min of consumption, and continued increasing through at least 280 min post consumption (the last time point sampled) compared to a placebo control [46].

The importance of the microbiota to D-xylose disappearance is verified by studies with germ-free or antibiotic-treated mice and rats [50–53]. Conventional rats had 87.8% total tract D-xylose disappearance compared to 74.3% in germ-free rats, with all differences occurring in the cecum and colon [51]. Studies with wild-caught rock mice (*Aethomys namaquensis*) who consume xylose containing nectar showed a 43% reduction in xylose metabolism following a heavy antibiotic regimen to drastically reduce gut microbial populations [52, 53]. The xylose-metabolizing gut bacteria were identified as an inducible population with higher numbers present when xylose is present in the diet [52]. If a xylose-metabolizing hindgut population is inducible in pigs, it is possible that the extent to which xylose is fermented may increase as pigs are adapted to diets with higher free xylose concentrations.

Xylose oligomers hydrolyzed from arabinoxylans in the small intestine by xylanase can be readily fermented by microbial populations in the distal ileum, cecum, and colon [27, 49, 54]. Xylose utilization was demonstrated by pig cecal cultures which fermented xylose at the same rate and curve of gas production as glucose fermentation [49]. Xylose fermentation generally results in greater acetate and butyrate proportions compared to glucose fermentation [49, 54, 55]. These data indicate that the ability of pig microbiota to utilize xylose is not limited. Data in rock mice indicate the xylose-fermenting bacterial population may be inducible with increased dietary D-xylose concentration [52].

## Xylose metabolism

Distinguishing the mechanism by which xylose disappears from the small intestine, either by absorption of the intact sugar or by microbial fermentation, is essential for determining its value to the pig in terms of energy metabolism. Very little is known about xylose metabolic utilization in vertebrates but a large majority of dietary supplemented D-xylose consistently appears in the urine [15, 27, 29] and significantly decreases the metabolizable energy (ME) available from the diet [20, 21, 28, 39]. For most carbohydrates, fermentation is a less efficient means of releasing dietary energy as compared with direct absorption and oxidation; however, in the case of xylose, it can be argued that fermentation may be more efficient.

In all species, urinary xylose excretion increases as dietary D-xylose inclusion increases [16, 21, 27, 39, 56]. Dietary D-xylose supplementation at 0.2% of DM resulted in 35% being excreted in the urine [28], while 10% D-xylose inclusion resulted in 37 – 45% urinary excretion [21, 27], and at 20% dietary D-xylose, 52.6% was excreted in urine [27]. The finding that the proportion of xylose excreted in the urine increases with higher intake suggests there may be some type of threshold for xylose metabolic capacity and that the energy potentially derived from xylose metabolism may change with the amount absorbed.

The detrimental effect of D-xylose on diet ME may be due to more than increased urinary xylose excretion. Verstegen et al. [21] compared ME values in pigs fed diets with 10% D-xylose or 5% D-glucose. Metabolizable energy was significantly lower in the xylose diets (2,819 vs. 2,924 kJ/d) and the difference was solely due to greater energy excreted in the urine (308 kJ/d vs. 88 kJ/d, for xylose and glucose, respectively). Surprisingly, the extra urinary energy from the xylose diet was more than could be accounted for by the presence of xylose alone. Non-xylose energy in the urine was double that of the control pigs [21]. Similarly, differences in urinary excretion of xylose between pigs fed either 10% or 20% D-xylose were not completely reflected in urinary excretion of energy [27]. Urinary xylose metabolites, such as threitol, were not measured in these experiments but may have contributed to the energetic differences.

Heat production data determined through indirect calorimetry helps to clarify when and how xylose may be altering energy metabolism in the pig. Pigs dosed with 500 g of D-xylose had increased heat production and the majority of xylose urine excretion was on the day of dosing, with only 2 - 6 g excreted on the following day [28]. Yet, the increase in heat production persisted until the third day after xylose dosing [28]. The authors proposed that this reflected either delayed metabolism of xylose or an increase in fermentation. These data provide further evidence of the pig’s capacity to convert xylose into energy, although the efficiency may be limited. The relative contribution of fermentation to the energy balance remains unclear.

Clearly, xylose does not contribute to ME with the same efficiency as glucose when provided as a purified monomer at high dietary concentrations (5 - 40%). It has yet to be determined if this effect holds for xylose monomers hydrolyzed in vivo from arabinoxylans resulting from inclusion of xylanase and supporting enzymes in the diet. The concentration of xylose released would depend on dietary NSP composition and carbohydrase efficiency, which is not frequently reported. Other than quantifying its contribution to ME, experiments in pigs have not examined further metabolic effects of increased xylose absorption.

Few data are available regarding xylose metabolism in pigs, but relevant studies in other monogastric species help to clarify the potential metabolic impact of xylose. Dietary xylose supplementation in rats has been shown to increase blood glucose levels over time, but not liver glycogen [57, 58]. Intravenous infusions of D-xylose solutions (10 - 20 g over 10 min) in humans also increased blood glucose concentrations and resulted in a drop in serum inorganic phosphate concentration [56]. This research seemed to indicate xylose conversion to glucose, a phenomenon not supported by current research. However, if xylose can be oxidized to CO_2_ for energy production or metabolized through the pentose phosphate pathway, it is possible that glucose may be spared.

Xylose oxidation to CO_2_ has been demonstrated in humans; infused D-xylose-1-^14^C appeared as expired ^14^CO_2_ in humans within 15 min [56]. Expired CO_2_ was maximally labeled 45 min following the end of D-xylose-1-^14^C infusion; the label was detectable for 6 h, and cumulatively represented 15.5% of the administered label [56]. This study was the first to demonstrate metabolism of xylose carbon separate from microbial fermentation.

Similarly, guinea pigs have been shown to oxidize xylose to CO_2_ [59]. Expired CO_2_ was maximally labeled 75 min after intraperitoneal injection of D-xylose-1-^14^C, with total ^14^C recovery representing 11.3% of the injected dose. About 50% of the xylose was excreted in the urine with the vast majority appearing within 5 h after injection [59]. Results from this thorough experiment indicate the ability of multiple tissues to absorb xylose. The distribution of ^14^C retained in tissues was greatest in the muscle followed by spleen, pancreas, liver, heart, and was lowest in the kidney. However, when slices of the tissues were measured for the oxidation of D-xylose-1-^14^C to ^14^CO_2_, the kidney was the most active followed by the liver, pancreas, spleen, heart, diaphragm, and lastly muscle [59]. Kidney tissue oxidized 164 times more D-xylose-1-^14^C than muscle.

Furthermore, D-xylose-1-^14^C metabolism was compared between control, intact animals and nephrectomized guinea pigs [59]. Control animals oxidized 10.8% of the injected dose to ^14^CO_2_ in 4 h with 41.3% excreted in urine and 47.9% unrecovered. Nephrectomy increased ^14^CO_2_ excretion 2- to 3-fold with about 75% of the injected dose unrecovered [59]. However, the capacity may have been even greater considering that the kidneys significantly contributed to xylose oxidation; therefore, their removal may have reduced the overall capacity to oxidize xylose. In sum, these results provide evidence for xylose oxidation to CO_2_ in multiple tissues, with the greatest capacity in the kidney. Some absorbed D-xylose may be retained in tissues throughout the body for longer than 6 h, and the capacity for greater xylose oxidation to CO_2_ exists if no other means of excretion are available [59].

These data provide convincing evidence that multiple species are capable of metabolizing at least some absorbed xylose to CO_2_ [56, 59]. Evidence thus far seems to point to critical roles of the liver and kidneys. Severe liver dysfunction had little impact in human xylose absorption tests, indicating that hepatic tissue is not significantly involved [29]. Furthermore, xylose supplementation to fasted rats did not increase liver glycogen concentration compared to fasted controls [60], indicating that the liver cannot metabolize xylose to glucose for storage as glycogen.

**Fig. 3:**
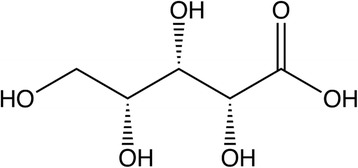
D-xylonic acid

Current understanding of metabolic pathways in pigs and other monogastric animals support two possible routes for D-xylose metabolism. The first is oxidation by D-xylose dehydrogenase (EC 1.1.1.175) to D-xylonic acid (Fig. 3) via a D-xylonolactone intermediate. D-xylose dehydrogenase has been identified and purified in pig liver [61] as well as in monkey kidney, dog liver, and rabbit lens [61, 62]. In vitro data from guinea pigs supports the presence of pentose dehydrogenase activity in liver extracts which is separate and distinct from dehydrogenases for glucose [59]. In liver extracts, D-xylose-1-^14^C was oxidized to D-xylonic acid-1-^14^C and D-xylonic acid-1-^14^C injected in vivo was also oxidized to ^14^CO_2_ [59]. These data support that xylose oxidation to CO_2_ can involve conversion to xylonic acid and subsequent decarboxylation [59, 61, 62].

**Fig. 4:**
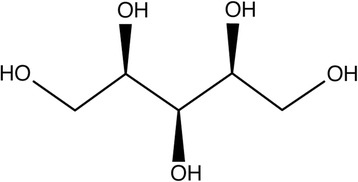
D-xylitol

A second potential pathway is the reduction of D-xylose by aldose reductase (EC 1.1.1.21) to D-xylitol (Fig. 4) which is then converted to D-xylulose and can be metabolized through the pentose phosphate pathway. Aldose reductase exhibits broad substrate specificity, including D-xylose and a variety of sugars [63]; expression of aldose reductase genes has been measured in pig lens [64], spleen, lung, ovary, adrenal, endometrium, kidney, and liver tissues [64, 65]. Xylose metabolism through either of these two potential pathways is possible; however, efficiency of the reactions depends on the distribution, abundance, and activity of D-xylose dehydrogenase and aldose reductase in pig tissues, something which has not yet been fully described.

**Fig. 5:**
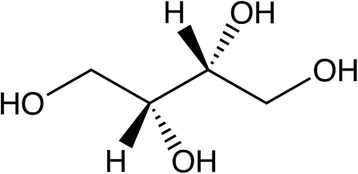
D-threitol

Xylose can also be metabolized to threitol, a urinary metabolite (Fig. 5). It has been measured in urine following D-xylose consumption by humans [66] and pigs [21]; human data seem to indicate a role for the liver. In healthy patients dosed with D-xylose, 15% of the xylose excreted in the urine was actually recovered as D-threitol within 5 h post-dosing; this proportion increased when the collection time was extended to 24 h [66]. Delayed excretion is not observed with xylose measured in the urine and seems to indicate a delayed conversion to and excretion of threitol. Threitol excretion was decreased in patients with cirrhotic liver disease which indicates a substantial portion of the xylose to threitol conversion may occur in the liver [66]. Furthermore, similar amounts of urinary threitol were noted after both intravenous and oral administration of xylose, supporting the hypothesis that the conversion is occurring in the liver as opposed to the intestines [66].

Although specific pathways of xylose metabolism have not yet been clarified, results from more recent studies provide information on the effects of xylose supplementation on metabolism of other nutrients and overall physiology. Hepatic expression of enzymes and transcription factors involved in glucose and lipid metabolism was measured in broiler chickens fed diets containing 0%, 5%, or 15% D-xylose [19]. The expression of phosphoenol pyruvate carboxykinase (PEPCK), a key enzyme in gluconeogenesis, did not appear to be affected by xylose supplementation at any time point up to 300 min post ingestion [19]. However, in poultry, the kidney is the major organ for gluconeogenesis [48] and treatment differences may have been observed if kidney samples had been analyzed for this enzyme instead. In the fasted state, hepatic pyruvate carboxylase was linearly increased by xylose [19]. Because pyruvate carboxylase is a rate limiting enzyme for hepatic gluconeogenesis which is active when glycogen stores are depleted, such as occurs in starvation, this result corroborates reports that xylose depletes hepatic glycogen stores in poultry [18, 39].

Understanding the effect of xylose on glucose metabolism is integral to understanding its contribution to overall energy metabolism. Potential effects must be gleaned from studies on other monogastric species as no data are available in pigs. Rats have been used as a model to evaluate potential xylose anti-diabetic effects; in one study, they were fed meals containing 5% or 10% D-xylose for 14 d [67]. In agreement with the attenuated glycemic effects discussed earlier, xylose supplementation decreased fasting blood glucose in a dose-dependent manner and decreased hepatic glycogen concentrations [67]. In contrast to the data in poultry, hepatic PEPCK protein levels were reduced in xylose supplemented rats indicating lower rates of gluconeogenesis. In vitro, xylose also increased insulin secretion from pancreatic β-cells [67]. These data indicate xylose is unlikely to contribute substantially to overall energy balance in pigs; in high enough concentrations, xylose may affect postprandial glucose metabolism [19, 67].

Xylose supplementation may also affect lipid metabolism. D-xylose supplementation at 5% and 10% of a high-fat diet reduced weight gain, improved serum lipid profiles and reduced hepatic lipid accumulation in obese mice [47]. These effects appeared to be influenced by changes in the expression of genes that mediate adipocyte differentiation, lipogenesis, and β-oxidation of fatty acids in adipose tissues. Xylose supplementation was associated with lower mRNA levels of sterol regulatory element-binding protein 1C, fatty acid synthase, adipocyte marker, and CCAAT/enhancer-binding protein-α in visceral adipose tissue of mice [47]. In the liver, xylose supplementation decreased protein levels of fatty acid synthase and peroxisome proliferator-activated receptor-γ by at least 40% compared to the high-fat diet control group [47]. These results indicate that xylose supplementation may suppress lipogenesis, and may give insight into mechanisms through which dietary xylose levels greater than 5% reduce ADG in pigs and poultry.

In the muscle, xylose may not be metabolized directly or efficiently for energy [59], but it does appear to at least be transported into the muscle cell [59]; and this transportation is increased in the presence of insulin [68]. Additionally, xylose may influence myocyte glucose transport. High levels of xylose were found to upregulate the glucose transport system [69] and generally increase glucose uptake by mouse myotubes [67]. Gruzman et al. [69] reported that xylose did not utilize glucose transporters to enter myotubes but suggested the presence of a highly specific pentose transport system in skeletal muscles. These data corroborate earlier reports of xylose retention in muscle [59], yet the transport mechanism and eventual metabolic fate remain unclear.

While the ambiguity surrounding xylose metabolism is frustrating from a nutritional perspective, it is not surprising from an evolutionary perspective. Monogastric animals do not have endogenous enzymes capable of degrading xylan to release monomeric xylose for absorption in the small intestine; consequently, there was little need to develop efficient mechanisms for xylose metabolism. Furthermore, xylose is a rare carbohydrate in mammalian cells and so far is only found as a link between the protein and glycosaminoglycan chains of some proteoglycans and in the Notch receptor [1]. The Notch receptor is part of a highly conserved core signaling pathway required for various cell fate decisions at multiple stages of development [70]. However, UDP-xylose, the activated precursor for xylose involvement in these structures, is synthesized from UDP-glucose and is not derived from dietary xylose sources [1]. As a result, the absence of an efficient or well-conserved pathway for xylose metabolism comes as no surprise because there has been no need. In practical terms, it also means that biochemists are not likely to find consistent mechanisms of xylose metabolism across species. Nor would nutritionists be highly motivated to evaluate the specific effects of xylose supplementation or xylose release from enzyme hydrolysis. One consistency regarding xylose metabolism, however, is the ability of intestinal microbiota to ferment xylose and arabinoxylan. Given the inefficiency of xylose metabolism when absorbed intact by enterocytes, a critical comparison with the energy derived from xylose fermentation is necessary to determine the most energetically efficient use of dietary xylose.

## Conclusions

Xylose appears to be poorly utilized by monogastric animals, but xylose metabolism to CO_2_ is possible. Increased dietary concentrations of D-xylose linearly decreases ADG, feed intake, and feed efficiency in pigs. Even though xylose disappears almost completely from the small intestine, a high proportion of what is absorbed is excreted in the urine (35 – 50%), either as xylose or as a metabolite. Of the portion retained in the body, only a small percentage is fully oxidized to CO_2_. This indicates that xylose is unlikely to contribute in any significant manner to energy balance in the pig through oxidative pathways. Its energetic contribution through fermentation may be less efficient, but quantitatively more important. However, this hypothesis has yet to be tested. More specifically, xylose has been reported to inhibit sucrase activity leading to decreased post-prandial blood glucose and insulin levels; further, xylose decreases the expression of genes involved in lipogenesis. Combined, these effects are beneficial to humans, especially for those with diabetes, but are negative in growing pigs.

It is important to consider that only one study has been reported using dietary concentrations of D-xylose below 5% in pigs [28]. It has yet to be determined if dietary concentrations more relevant to swine nutrition (i.e. 1 - 4% xylose) would exert similar effects. It must also be determined if pigs develop increased capacity for xylose metabolism as adaptation time to diets supplemented with xylose or xylanase increases. Data regarding xylose absorption support the hypothesis that mechanisms exist to handle low concentrations of xylose, but those mechanisms may be easily overwhelmed as luminal concentrations increase. It is possible that these mechanisms may be up-regulated as the pig adapts to higher luminal xylose concentrations. Furthermore, the effects of xylose on nitrogen balance and muscle metabolism have yet to be evaluated. Lastly, there is a need for a more comprehensive understanding of the action and hydrolysis products of exogenous xylanase in vivo, an understanding of how pig tissues metabolize hydrolyzed xylose monomers, and clarity on the contribution of microbial xylose fermentation to dietary xylose absorption and the energy value of xylanase supplemented diets.

## Abbreviations

ADG: Average daily gain
DM: Dry matter
GLP-1: Glucagon like peptide-1
ME: Metabolizable energy
NSP: Non-starch polysaccharide
PEPCK: Phosphoenol pyruvate carboxykinase
SCFA: Short chain fatty acids
